# Prevalence and Characteristics of Autism Spectrum Disorder Among Children Aged 8 Years — Autism and Developmental Disabilities Monitoring Network, 11 Sites, United States, 2018

**DOI:** 10.15585/mmwr.ss7011a1

**Published:** 2021-12-03

**Authors:** Matthew J. Maenner, Kelly A. Shaw, Amanda V. Bakian, Deborah A. Bilder, Maureen S. Durkin, Amy Esler, Sarah M. Furnier, Libby Hallas, Jennifer Hall-Lande, Allison Hudson, Michelle M. Hughes, Mary Patrick, Karen Pierce, Jenny N. Poynter, Angelica Salinas, Josephine Shenouda, Alison Vehorn, Zachary Warren, John N. Constantino, Monica DiRienzo, Robert T. Fitzgerald, Andrea Grzybowski, Margaret H. Spivey, Sydney Pettygrove, Walter Zahorodny, Akilah Ali, Jennifer G. Andrews, Thaer Baroud, Johanna Gutierrez, Amy Hewitt, Li-Ching Lee, Maya Lopez, Kristen Clancy Mancilla, Dedria McArthur, Yvette D. Schwenk, Anita Washington, Susan Williams, Mary E. Cogswell

**Affiliations:** ^1^National Center on Birth Defects and Developmental Disabilities, CDC, Atlanta, Georgia; ^2^University of Utah School of Medicine, Salt Lake City, Utah; ^3^University of Wisconsin, Madison, Wisconsin; ^4^University of Minnesota, Minneapolis, Minnesota; ^5^University of Arkansas for Medical Sciences, Little Rock, Arkansas; ^6^University of California, San Diego, California; ^7^Rutgers New Jersey Medical School, Newark, New Jersey; ^8^Vanderbilt University Medical Center, Nashville, Tennessee; ^9^Washington University, St. Louis, Missouri; ^10^Johns Hopkins University, Baltimore, Maryland; ^11^University of Arizona, Tucson, Arizona; ^12^Oak Ridge Institute for Research and Education, Oak Ridge, Tennessee

## Abstract

**Problem/Condition:**

Autism spectrum disorder (ASD).

**Period Covered:**

2018.

**Description of System:**

The Autism and Developmental Disabilities Monitoring (ADDM) Network conducts active surveillance of ASD. This report focuses on the prevalence and characteristics of ASD among children aged 8 years in 2018 whose parents or guardians lived in 11 ADDM Network sites in the United States (Arizona, Arkansas, California, Georgia, Maryland, Minnesota, Missouri, New Jersey, Tennessee, Utah, and Wisconsin). To ascertain ASD among children aged 8 years, ADDM Network staff review and abstract developmental evaluations and records from community medical and educational service providers. In 2018, children met the case definition if their records documented 1) an ASD diagnostic statement in an evaluation (diagnosis), 2) a special education classification of ASD (eligibility), or 3) an ASD International Classification of Diseases (ICD) code.

**Results:**

For 2018, across all 11 ADDM sites, ASD prevalence per 1,000 children aged 8 years ranged from 16.5 in Missouri to 38.9 in California. The overall ASD prevalence was 23.0 per 1,000 (one in 44) children aged 8 years, and ASD was 4.2 times as prevalent among boys as among girls. Overall ASD prevalence was similar across racial and ethnic groups, except American Indian/Alaska Native children had higher ASD prevalence than non-Hispanic White (White) children (29.0 versus 21.2 per 1,000 children aged 8 years). At multiple sites, Hispanic children had lower ASD prevalence than White children (Arizona, Arkansas, Georgia, and Utah), and non-Hispanic Black (Black) children (Georgia and Minnesota). The associations between ASD prevalence and neighborhood-level median household income varied by site. Among the 5,058 children who met the ASD case definition, 75.8% had a diagnostic statement of ASD in an evaluation, 18.8% had an ASD special education classification or eligibility and no ASD diagnostic statement, and 5.4% had an ASD ICD code only. ASD prevalence per 1,000 children aged 8 years that was based exclusively on documented ASD diagnostic statements was 17.4 overall (range: 11.2 in Maryland to 29.9 in California). The median age of earliest known ASD diagnosis ranged from 36 months in California to 63 months in Minnesota.

Among the 3,007 children with ASD and data on cognitive ability, 35.2% were classified as having an intelligence quotient (IQ) score ≤70. The percentages of children with ASD with IQ scores ≤70 were 49.8%, 33.1%, and 29.7% among Black, Hispanic, and White children, respectively. Overall, children with ASD and IQ scores ≤70 had earlier median ages of ASD diagnosis than children with ASD and IQ scores >70 (44 versus 53 months).

**Interpretation:**

In 2018, one in 44 children aged 8 years was estimated to have ASD, and prevalence and median age of identification varied widely across sites. Whereas overall ASD prevalence was similar by race and ethnicity, at certain sites Hispanic children were less likely to be identified as having ASD than White or Black children. The higher proportion of Black children compared with White and Hispanic children classified as having intellectual disability was consistent with previous findings.

**Public Health Action:**

The variability in ASD prevalence and community ASD identification practices among children with different racial, ethnic, and geographical characteristics highlights the importance of research into the causes of that variability and strategies to provide equitable access to developmental evaluations and services. These findings also underscore the need for enhanced infrastructure for diagnostic, treatment, and support services to meet the needs of all children.

## Introduction

Autism spectrum disorder (ASD) is a developmental disability that can cause a wide range of challenges in social interaction, communication, and behavior. The *Diagnostic and Statistical Manual of Mental Disorders, 5th Edition* (*DSM-5*) defines ASD as the occurrence of persistent impairments in social interaction and the presence of restricted, repetitive patterns of behaviors, interests, or activities (*1*). CDC began monitoring the prevalence of ASD in 1996, initially conducting studies among children in metropolitan Atlanta, Georgia (*2*). The Children’s Health Act of 2000 authorized CDC to establish the Autism and Developmental Disabilities Monitoring (ADDM) Network. Since 2000, CDC has supported biennial surveillance to track ASD prevalence in multiple communities.

ASD prevalence estimates have increased from 6.7 (one in 150) per 1,000 children aged 8 years at ADDM Network sites in surveillance years 2000 and 2002 to 18.5 (one in 54) in surveillance year 2016 (*3*–*10*). Over time, the proportion of children with ASD who also have intellectual disability has decreased from approximately one half in 2000 and 2002 to one third in 2016 (*3*,*4*,*10*). The ADDM Network also has reported decreasing racial and ethnic disparities in ASD prevalence, recently describing no overall difference in ASD prevalence between non-Hispanic White (White) and non-Hispanic Black (Black) children aged 8 years according to 2016 ADDM data (*10*). However, other disparities have remained unchanged. Black children with ASD were more likely to have intellectual disability than White children with ASD, Black children with ASD were first evaluated at older ages than White children with ASD, and the overall ASD prevalence among Hispanic children was lower than among Black and White children (*10*). These findings suggest disparities in access to identification of and services for ASD across groups or communities.

This report provides updated data on ASD prevalence and characteristics among children aged 8 years from 11 ADDM Network sites in 2018, including prevalence by site, sex, race and ethnicity, and neighborhood socioeconomic status (SES). Children with ASD also are classified in terms of co-occurring intellectual disability (on the basis of cognitive test data), the number identified in medical and educational settings, median ages at first evaluation, and median ages at diagnosis. Health care and service providers, educators, researchers, and policymakers can use ADDM Network data to inform equitable allocation of services and support for children with ASD and their families.

## Methods

### Surveillance Sites and Procedures

For 2018, the ADDM Network consisted of 11 sites (Arizona, Arkansas, California, Georgia, Maryland, Minnesota, Missouri, New Jersey, Tennessee, Utah, and Wisconsin). Sites were competitively funded, and each selected a contiguous geographic area of its state to monitor ASD among children aged 8 years (Table 1). Children included in the 2018 ADDM Network data were born in 2010 and had a parent or guardian who lived in surveillance areas of the 11 sites during 2018. All sites functioned as public health authorities under the Health Insurance Portability and Accountability Act of 1996 Privacy Rule and met applicable local institutional review board, privacy, and confidentiality requirements under 45 CFR 46 (*11*).

**TABLE 1:** Surveillance sites and data sources used for surveillance in each site — Autism and Developmental Disabilities Monitoring Network, 11 sites, United States, 2018

Site	Surveillance area description	Total population aged 8 yrs	% Male	% White, non-Hispanic	% Black, non-Hispanic	% Hispanic	% Asian/Pacific Islander	% American Indian/Alaska Native	Types of data sources used	Education data sources (% population coverage)*	% of requested records fully accessible for chart review
Arizona	Part of one county in metropolitan Phoenix	13,313^†^	51.1	42.7	7.2	42.9	3.8	3.4	Health, education	100	100
Arkansas	21 counties in central Arkansas	15,435	51.5	63.6	25.0	9.5	1.4	0.4	Health, education	100	98.1
California	Part of one county in metropolitan San Diego	15,076^†^	50.9	24.3	9.5	51.8	14.0	0.4	Health, education, state developmental disability services	100	99.8
Georgia	Two counties in metropolitan Atlanta	23,580	50.9	25.9	40.5	24.5	8.9	0.2	Health, education	100	66.6
Maryland	Five counties in suburban Baltimore	20,666	50.7	55.3	25.8	8.9	9.8	0.3	Health, education	100	31.4
Minnesota	Parts of three counties in the Twin Cities metropolitan area	10,081^†^	51.2	51.1	24.7	14.3	8.3	1.7	Health, education	100	99.9
Missouri	Five counties in metropolitan St. Louis	24,481	51.3	65.6	25.2	5.1	3.8	0.2	Health	0^§^	100
New Jersey	Part of two counties in New York metropolitan area	17,289^†^	51.5	28.0	32.3	33.8	5.6	0.3	Health, education	100	99.7
Tennessee	11 counties in middle Tennessee	25,237	51.2	62.8	19.6	13.9	3.3	0.3	Health, education	100	85.4
Utah	Three counties in northern Utah	25,459	51.3	71.1	2.5	21.2	4.5	0.6	Health, education, early intervention	100	67.7
Wisconsin	Eight counties in southeastern Wisconsin	29,664	50.9	57.5	19.4	17.4	5.2	0.5	Health, education, Medicaid claims, state-funded long-term care program	24.1	100
**Total**		**220,281**	**51.1**	**51.6**	**21.2**	**20.5**	**6.0**	**0.6**			**87.1**

* For public schools in the surveillance area.

^†^ Denominator excludes school districts that were not included in the surveillance area, calculated from National Center for Education Statistics enrollment counts of third graders during the 2018–19 school year.

^§^ Education data at this site could be collected if they were included in a child’s medical or service records.

### Case Ascertainment and Surveillance Case Definition

The ADDM Network is an active records-based surveillance program using multiple sources of information within a community (Table 1). For surveillance year 2018, the ADDM Network adopted a case definition and data collection process and method to fit the increased availability of ASD diagnostic information in health and education records (*12*). As with the previous methods, which were based on the model created by CDC’s Metropolitan Atlanta Developmental Disabilities Surveillance Program (*13*), sites request records (electronic and paper-based) from community medical, education, and service providers containing specific special education exceptionalities or billing codes from the *International Classification of Diseases, Ninth Revision* (ICD-9) or *International Classification of Diseases, Tenth Revision* (ICD-10). Recommended ICD codes were described previously (*10*). All ADDM Network sites used records from medical service providers that evaluated children with developmental disabilities; however, the Missouri and Wisconsin sites did not have complete access to education records (Table 1). ADDM Network sites received information (including demographic data and ICD or special education codes) for children with one or more of the requested codes, and ADDM staff manually reviewed the contents of records. If any part of a child’s record indicated that the ASD case definition had been met, ADDM staff abstracted information from the child’s developmental evaluations, special education plans, and other documents (e.g., cognitive or IQ tests) and combined records across data sources. At certain sites, full record review could not be completed for all records due to COVID-19 pandemic restrictions on physically accessing the location where certain records were stored (Table 1).

Children met the ASD case definition if they were aged 8 years in 2018 (born in 2010), lived in the surveillance area for at least one day during 2018, and had documentation in health, service, or education records that they had ever received any of the following: 1) a written statement from a qualified professional (Supplementary Box, https://stacks.cdc.gov/view/cdc/111176) diagnosing ASD, 2) a special education classification of autism (either primary exceptionality of ASD or an evaluation concluding criteria for autism eligibility was met) in public school, or 3) an ASD ICD code (ICD-9 codes between 299.00 and 299.99 or ICD-10 codes in the F84 range except for F84.2) obtained from administrative or billing information. Six children with an ICD code for F84.2 (Rett syndrome) had no other indicators of ASD and did not meet the ASD case definition. ASD-related diagnostic conclusions (including instances when ASD was suspected or ruled out) were recorded verbatim from evaluations and were reviewed and confirmed by ADDM Network staff with clinical expertise at each site.

### Additional Data Sources and Variable Definitions

Population denominators were obtained from the National Center for Health Statistics vintage 2019 bridged-race postcensal population estimates for 2018 (*14*). Surveillance areas at four sites (Arizona, California, Minnesota, and New Jersey) comprised subcounty school districts, and public school enrollment counts were used to adjust the county population estimates described previously (*10*). When possible, sites linked data from children identified with ASD to birth certificate information from their state to obtain additional demographic information. Information about race and ethnicity was abstracted primarily from the medical or education records and, when missing, was augmented by birth certificate, administrative, or billing information. Children with race coded as other or multiracial were excluded from race-specific prevalence estimates, and the denominator data do not include those categories. Estimates for non-Hispanic American Indian/Alaska Native (AI/AN) children were not reported in most results because of small numbers.

Neighborhood-level SES was measured by median household income (MHI) at the census-tract level using the 2018 American Community Survey 5-year estimates (*15*). Census-tract–level population counts of children aged 8 years were estimated by dividing the number of children aged 5–9 years by five for each census tract. The census tracts included in the surveillance areas were classified into three approximately equal-sized population groups (i.e., tertiles) of low, medium, and high MHI on the basis of all sites combined. Children meeting the ADDM Network case definition for ASD were geocoded and assigned to an SES group corresponding to their 2018 address. Census tract information was available for 93.6% of children; the remainder were determined as living in the surveillance area on the basis of services receipt or school attendance in 2018 indicating residence within the surveillance area but precluding identification of residential census tract.

Age at first developmental evaluation was limited to children with information on the earliest collected or historically reported evaluation (including reports of previous ASD-related diagnoses) available. Age at earliest evaluation also was calculated using the same approach as previous ADDM Network surveillance reports. Age at first ASD diagnosis was based on the earliest documented age when a qualified professional diagnosed ASD in a child or reported when another provider diagnosed ASD. Intellectual disability status was based on IQ scores ≤70 on a child’s most recent cognitive test or a statement from a qualified professional about a child’s cognitive ability in a developmental evaluation.

### Analytic Methods

Overall ASD prevalence estimates included all children who met the case definition from the 11 sites. Prevalence was calculated as the number of children with ASD divided by the total number of children in the defined population or group per 1,000 children. Prevalence was calculated overall, by sex, and by race and ethnicity for White, Black, Hispanic, Asian/Pacific Islander (A/PI), and AI/AN children. The Wilson score method was used to calculate 95% confidence intervals (CIs). Pearson chi-square tests were used to compare proportions, and the Mantel-Haenszel (Woolf) test of homogeneity compared prevalence ratios across sites. Permutation tests were conducted to test differences in medians. Cochran Armitage tests were used to detect trends in prevalence across SES tertiles. Prevalence estimates with a relative standard error >30% (and ratios calculated from those estimates) were considered to have limited statistical precision and were suppressed. Statistical tests with p values <0.05 and prevalence ratio 95% CIs that excluded 1.0 were considered statistically significant. R software (version 4.5; R Foundation) and additional packages were used to conduct analyses. Additional information about the statistical software is available (Supplementary Table 1, https://stacks.cdc.gov/view/cdc/111176).

## Results

### ASD Prevalence

The overall ASD prevalence per 1,000 children aged 8 years was 23.0 and ranged from 16.5 in Missouri to 38.9 in California (Table 2). The overall male-to-female prevalence ratio was 4.2, and site-specific ratios ranged from 3.3 to 5.2.

**TABLE 2:** Prevalence* of autism spectrum disorder among children aged 8 years, overall and by sex — Autism and Developmental Disabilities Monitoring Network, 11 sites, United States, 2018

Site	Overall^†^			Male prevalence (95% CI)	Female prevalence (95% CI)	Male-to-female prevalence ratio (95% CI)^§^
	No. with ASD	Total population	Prevalence (95% CI)			
Arizona	331	13,313	24.9 (22.4–27.6)	37.9 (33.6–42.7)	11.2 (8.9–14.1)	3.4 (2.6–4.4)
Arkansas	353	15,435	22.9 (20.6–25.3)	36.7 (32.8–41.1)	8.1 (6.3–10.4)	4.5 (3.4–5.9)
California	586	15,076	38.9 (35.9–42.1)	64.4 (59.2–70.2)	12.3 (10.0–15.1)	5.2 (4.2–6.5)
Georgia	514	23,580	21.8 (20.0–23.7)	35.2 (32.1–38.7)	7.8 (6.3– 9.5)	4.5 (3.6–5.7)
Maryland	423	20,666	20.5 (18.6–22.5)	33.1 (29.8–36.7)	7.5 (6.0– 9.3)	4.4 (3.5–5.7)
Minnesota	277	10,081	27.5 (24.5–30.9)	43.7 (38.5–49.7)	10.4 (7.9–13.6)	4.2 (3.1–5.7)
Missouri	405	24,481	16.5 (15.0–18.2)	25.0 (22.4–27.9)	7.6 (6.2– 9.4)	3.3 (2.6–4.1)
New Jersey	491	17,289	28.4 (26.0–31.0)	45.6 (41.4–50.1)	10.1 (8.2–12.5)	4.5 (3.6–5.7)
Tennessee	573	25,237	22.7 (20.9–24.6)	36.0 (32.9–39.3)	8.8 (7.3–10.6)	4.1 (3.3–5.1)
Utah	548	25,459	21.5 (19.8–23.4)	33.1 (30.2–36.3)	9.3 (7.7–11.1)	3.6 (2.9–4.4)
Wisconsin	557	29,664	18.8 (17.3–20.4)	30.0 (27.4–32.8)	7.1 (5.9– 8.6)	4.2 (3.4–5.2)
**Total**	**5,058**	**220,281**	**23.0 (22.3–23.6)**	**36.5 (35.4–37.6)**	**8.8 (8.2– 9.4)**	**4.2 (3.9–4.5)**

**Abbreviations:** ASD = autism spectrum disorder; CI = confidence interval.

* Per 1,000 children aged 8 years.

^†^ All children are included in the total regardless of sex or race/ethnicity.

^§^ Wilson score 95% CIs exclude 1.0 in all sites, indicating significantly higher prevalence among males than among females; Mantel Haenszel (Woolf) test of homogeneity of prevalence ratios across sites, p = 0.15, indicating little heterogeneity in prevalence ratios across sites.

Overall ASD prevalence per 1,000 children aged 8 years was similar among White, Black, A/PI, and Hispanic children (21.2, 22.3, 22.2, and 22.5, respectively) (Table 3). Compared with Hispanic children, ASD prevalence was higher among White children in Arizona, Arkansas, Georgia, and Utah and higher among Black children in Georgia and Minnesota. ASD prevalence was lower among White children than Black children in Maryland and Minnesota. ASD prevalence among A/PI children differed from Black children in Georgia (Black-to-A/PI prevalence ratio: 1.4). Among AI/AN children, ASD prevalence was 29.0 per 1,000 overall; this was higher than among White children overall but not different from other racial and ethnic groups.

**TABLE 3:** Prevalence* of autism spectrum disorder among children aged 8 years, by race/ethnicity — Autism and Developmental Disabilities Monitoring Network, 11 sites, United States, 2018

Site	Prevalence (95% CI)				Prevalence ratio (95% CI)					
	White, non-Hispanic	Black, non-Hispanic	Hispanic	Asian/Pacific Islander	White, non-Hispanic to Black, non-Hispanic	White, non-Hispanic to Hispanic	Black, non-Hispanic to Hispanic	White, non-Hispanic to Asian/Pacific Islander	Black, non-Hispanic to Asian/Pacific Islander	Hispanic to Asian/Pacific Islander
Arizona	26.9 (23.0–31.5)	26.0 (17.6–38.0)	20.8 (17.4–24.9)	—^†^	1.0 (0.7–1.6)	1.3 (1.0–1.6)^§^	1.2 (0.8–1.9)	—^†^	—^†^	—^†^
Arkansas	23.8 (21.0–27.0)	19.2 (15.3–24.0)	14.9 (9.9–22.5)	—^†^	1.2 (1.0–1.6)	1.6 (1.0–2.5)^§^	1.3 (0.8–2.1)	—^†^	—^†^	—^†^
California	36.6 (31.0–43.2)	32.1 (24.2–42.6)	35.3 (31.5–39.6)	39.2 (31.8–48.4)	1.1 (0.8–1.6)	1.0 (0.8–1.3)	0.9 (0.7–1.2)	0.9 (0.7–1.2)	0.8 (0.6–1.2)	0.9 (0.7–1.1)
Georgia	23.3 (19.8–27.3)	24.0 (21.1–27.3)	11.4 (9.0–14.5)	16.6 (12.0–23.0)	1.0 (0.8–1.2)	2.0 (1.5–2.7)^§^	2.1 (1.6–2.8)^§^	1.4 (1.0–2.0)	1.4 (1.0–2.1)^§^	0.7 (0.5–1.0)
Maryland	15.6 (13.5–18.0)	24.8 (20.9–29.3)	19.1 (13.8–26.5)	19.2 (14.1–26.2)	0.6 (0.5–0.8)^§^	0.8 (0.6–1.2)	1.3 (0.9–1.9)	0.8 (0.6–1.1)	1.3 (0.9–1.8)	1.0 (0.6–1.6)
Minnesota	25.0 (21.1–29.7)	33.0 (26.6–40.7)	18.1 (12.4–26.4)	21.5 (13.6–33.7)	0.8 (0.6–1.0)^§^	1.4 (0.9–2.1)	1.8 (1.2–2.8)^§^	1.2 (0.7–1.9)	1.5 (0.9–2.5)	0.8 (0.5–1.5)
Missouri	17.8 (15.8–19.9)	14.3 (11.6–17.5)	—^†^	17.0 (10.5–27.5)	1.2 (1.0–1.6)	—^†^	—^†^	1.0 (0.6–1.7)	0.8 (0.5–1.4)	—^†^
New Jersey	24.0 (20.0–28.7)	25.6 (21.8–30.1)	30.1 (26.0–34.8)	29.7 (20.8–42.3)	0.9 (0.7–1.2)	0.8 (0.6–1.0)	0.9 (0.7–1.1)	0.8 (0.5–1.2)	0.9 (0.6–1.3)	1.0 (0.7–1.5)
Tennessee	20.6 (18.5–22.9)	24.1 (20.1–28.7)	25.9 (21.1–31.6)	22.5 (14.4–34.9)	0.9 (0.7–1.1)	0.8 (0.6–1.0)	0.9 (0.7–1.2)	0.9 (0.6–1.4)	1.1 (0.7–1.7)	1.2 (0.7–1.9)
Utah	22.3 (20.2–24.5)	—^†^	17.8 (14.6–21.7)	15.7 (10.0–24.7)	—^†^	1.3 (1.0–1.6)^§^	—^†^	1.4 (0.9–2.3)	—^†^	1.1 (0.7–1.9)
Wisconsin	18.0 (16.1–20.1)	16.5 (13.5–20.1)	21.3 (17.7–25.6)	15.0 (10.0–22.4)	1.1 (0.9–1.4)	0.8 (0.7–1.0)	0.8 (0.6–1.0)	1.2 (0.8–1.8)	1.1 (0.7–1.7)	1.4 (0.9–2.2)
**Total**	**21.2 (20.3–22.0)**	**22.3 (21.0–23.7)**	**22.5 (21.2–23.9)**	**22.2 (19.8–24.8)**	**0.9 (0.9–1.0)**	**0.9 (0.9–1.0)**	**1.0 (0.9–1.1)**	**1.0 (0.8–1.1)**	**1.0 (0.9–1.1)**	**1.0 (0.9–1.2)**

**Abbreviation:** CI = confidence interval.

* Per 1,000 children aged 8 years. (Overall American Indian/Alaska Native autism spectrum disorder prevalence per 1,000 was 29.0 [95% CI: 21.3–39.4]. Arizona was the only Autism and Developmental Disabilities Monitoring Network site meeting the threshold for statistical precision for American Indian/Alaska Native autism spectrum disorder prevalence; the site-specific prevalence per 1,000 was 33.0 [95% CI: 20.1–53.8].)

^†^ Estimate was suppressed because standard error for prevalence was ≥30% of estimate or prevalence ratio was based on an estimate that was suppressed.

^§^ 95% CI does not include 1.0.

**FIGURE 1:**
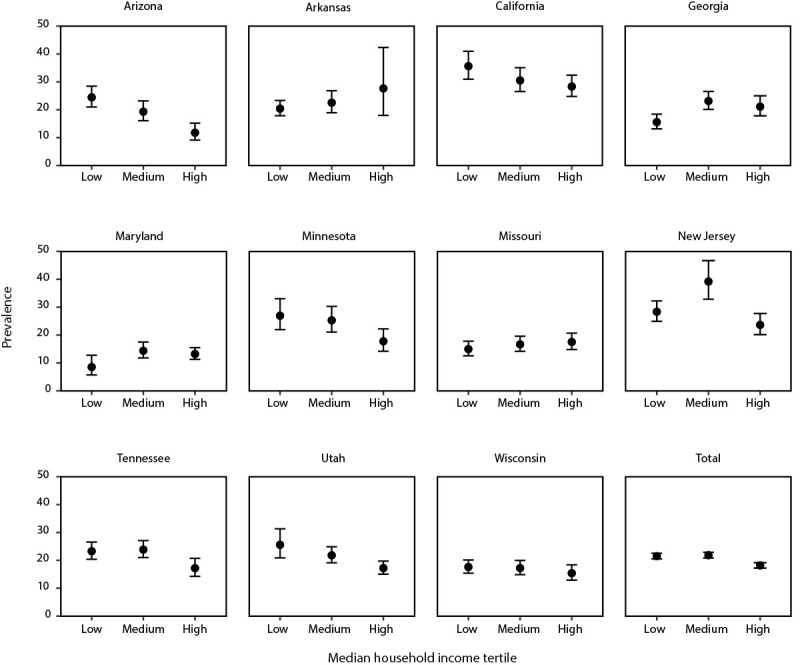
Prevalence* of autism spectrum disorder per 1,000 children aged 8 years, by median household income tertile and site† — Autism and Developmental Disabilities Monitoring Network, 11 sites, United States, 2018 * Dots are the point estimates and horizontal lines are the 95% confidence intervals. ^†^ Cochran Armitage test of trend results for association between socioeconomic status tertile and ASD prevalence, by site and overall: Arizona (p<0.001), Arkansas (p = 0.17), California (p = 0.03), Georgia (p = 0.01), Maryland (p = 0.21), Minnesota (p = 0.01), Missouri (p = 0.21), New Jersey (p = 0.15), Tennessee (p = 0.02), Utah (p<0.001), and Wisconsin (p = 0.27); all sites (p<0.001).

The association between census-tract–level MHI and ASD varied across sites (Figure 1). At five sites (Arizona, California, Minnesota, Tennessee, and Utah), a trend of lower ASD prevalence was observed among children living in census tracts with higher MHIs. At one site (Georgia), an association of higher ASD prevalence was found among children living in census tracts with higher MHIs. At five sites (Arkansas, Maryland, Missouri, New Jersey, and Wisconsin), no clear trend was found between ASD prevalence and MHI.

### ASD Identification

**FIGURE 2:**
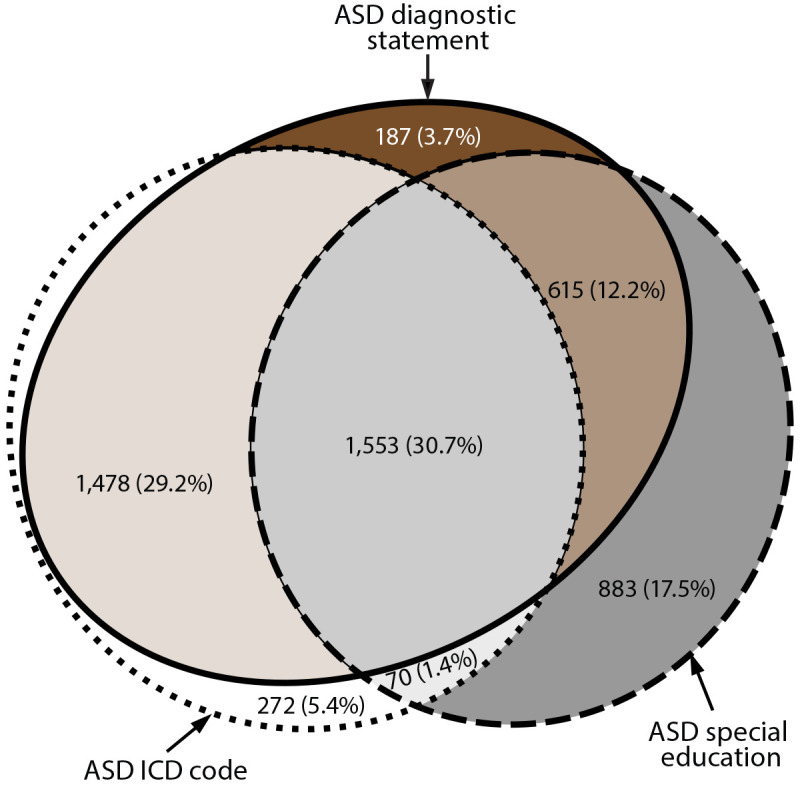
Euler diagram of different types of autism spectrum disorder identification among children aged 8 years with autism spectrum disorder* — Autism and Developmental Disabilities Monitoring Network, 11 sites, United States, 2018 **Abbreviations:** ASD = autism spectrum disorder; ICD = International Classification of Diseases. * N = 5,058.

Among the 5,058 children aged 8 years with ASD (i.e., who met the ASD case definition), 75.8% had a diagnostic statement of ASD documented in a developmental evaluation, 18.8% had an ASD special education classification or eligibility but did not have an ASD diagnostic statement, and 5.4% had an ASD ICD code only (Figure 2). Most (73.5%) children with ASD had at least two of the three types of ASD identification (e.g., an ASD diagnostic statement and an ASD ICD code). Among the 3,373 children with an ASD ICD code, 3,101 (91.9%) also had a documented ASD diagnostic statement or ASD special education classification. The percentage of children with ASD ascertained only through manual review of records is available (Supplementary Table 2, https://stacks.cdc.gov/view/cdc/111176).

**FIGURE 3:**
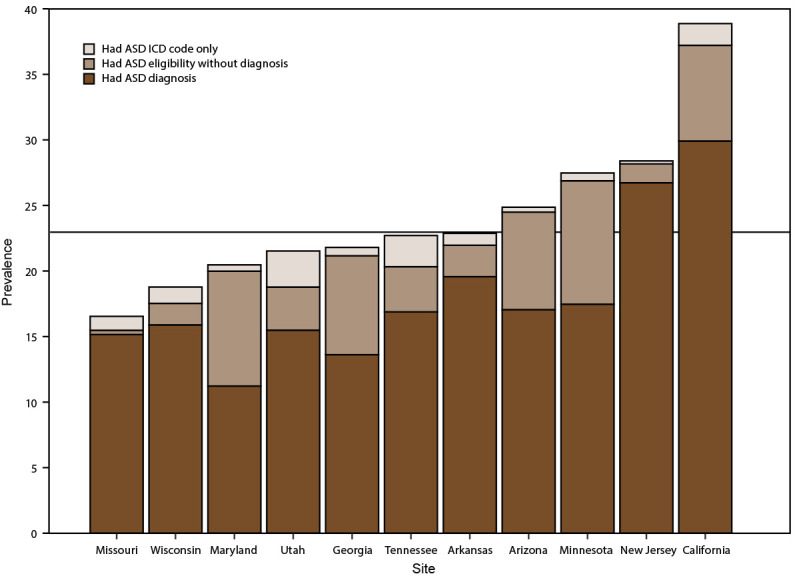
Prevalence* of autism spectrum disorder per 1,000 children aged 8 years, by identification type and site — Autism and Developmental Disabilities Monitoring Network, 11 sites, United States, 2018 **Abbreviations:** ASD = autism spectrum disorder; ICD = International Classification of Diseases. * Horizontal line is the overall Autism and Developmental Disabilities Monitoring Network prevalence of 23.0 per 1,000 children aged 8 years. Children with documented ASD statements could also have ASD classifications in special education or ASD ICD codes.

The proportion of children identified by diagnostic statements, special education eligibility, and ICD codes varied by site (Table 4). Across sites, the proportion of children with ASD who had a documented ASD diagnostic statement ranged from 54.8% in Maryland to 94.1% in New Jersey. ASD prevalence per 1,000 children aged 8 years that was based exclusively on documented ASD diagnostic statements was 17.4 overall (range: 11.2 in Maryland to 29.9 in California) (Figure 3). Arizona had the lowest proportion of children with ASD with an ASD ICD code (29.3%) and the second-highest proportion with an ASD special education classification (84.3%). In contrast, Missouri (a site without direct access to education sources) had the highest proportion of children with ASD with an ASD ICD code (94.6%) and the lowest with an ASD special education classification (26.4%).

**TABLE 4:** Autism spectrum disorder identification information among children aged 8 years meeting case definition, by site — Autism and Developmental Disabilities Monitoring Network, 11 sites, United States, 2018

Site	No. with ASD	Part of ASD case definition*			Evaluation in addition to meeting ASD case definition		
		% with an ASD ICD code	% with an ASD special education eligibility	% with an ASD diagnostic statement	% of all children with ASD with an evaluation summary diagnosis of suspected ASD	% of all children with ASD with an evaluation summary ever ruling out ASD (diagnosis or special education eligibility)^†^	% of all children with ASD ruled out (diagnosis or special education) more recently than documented ASD diagnosis or eligibility^†^
Arizona	331	29.3	84.3	68.6	37.8	15.4	4.8
Arkansas	353	67.7	66.3	85.6	48.7	15.3	4.5
California	586	58.7	88.2	77.0	20.0	24.7	12.5
Georgia	514	41.6	72.8	62.5	49.2	5.6	2.1
Maryland	423	53.9	72.6	54.8	32.4	11.8	4.5
Minnesota	277	61.0	82.3	63.5	18.4	10.8	3.6
Missouri	405	94.6	26.4	91.6	34.1	10.6	2.5
New Jersey	491	68.0	69.9	94.1	25.5	1.4	0.4
Tennessee	573	77.1	54.8	74.3	33.0	8.4	4.0
Utah	548	82.8	42.2	71.9	32.1	4.2	2.4
Wisconsin	557	84.2	33.6	84.6	34.1	12.9	2.5
**Total**	**5,058**	**66.7**	**61.7**	**75.8**	**33.1**	**10.9**	**4.1**

**Abbreviations:** ASD = autism spectrum disorder; ICD = International Classification of Diseases.

* ICD code, special education, and diagnosis can be interpreted as the individual sensitivity of each component related to the entire case definition.

^†^ Includes children who had ASD ruled out and had never had either a documented ASD diagnosis or special education exceptionality (i.e., had an ASD ICD code only).

Among children with ASD, a proportion had evaluation reports noting that ASD was suspected (but not confirmed) or was ruled out. Across sites, 33.1% of children with ASD had at one time suspected but not confirmed ASD. Overall, 10.9% of children with ASD had ever had an ASD diagnosis or special education eligibility ruled out (range: 1.4% in New Jersey to 24.7% in California). For most of these children, the classification or diagnosis of ASD was made after ASD had previously been ruled out; however, 4.1% (range: 0.4% in New Jersey to 12.5% in California) of all children with ASD had an evaluation ruling out ASD more recently than one confirming ASD.

### Cognitive Ability Among Children with ASD

The proportion of children aged 8 years with ASD and data on cognitive ability was 59.5% overall (range: 32.1% in Missouri to 88.7% in Arkansas) (Table 5). Among children with data on cognitive ability, the median age of the most recent cognitive test or examiner impression was 72 months (interquartile range: 56–89 months) (Supplementary Table 3, https://stacks.cdc.gov/view/cdc/111176). The proportions of girls and boys with ASD with data on cognitive ability did not significantly differ (56.8% versus 60.1%), whereas Black and White children were less likely than Hispanic children to have data on cognitive ability (54.8%, 58.2%, and 66.4%, respectively).

**TABLE 5:** Availability and distribution of intelligence quotient scores among children aged 8 years with autism spectrum disorder, by site, sex, and race/ethnicity — Autism and Developmental Disabilities Monitoring Network, 11 sites, United States, 2018

Site/Characteristic	Total no. with ASD	With IQ information	Cognitive level		
		No. (%)	IQ ≤70 (%)	IQ = 71–85 (%)	IQ >85 (%)*
**Site**					
Arizona	**331**	290 (87.6)	31.4	23.8	44.8
Arkansas	**353**	313 (88.7)	39.9	25.2	34.8
California	**586**	454 (77.5)	20.5	25.6	54.0
Georgia	**514**	353 (68.7)	38.5	18.4	43.1
Maryland	**423**	141 (33.3)	44.7	17.7	37.6
Minnesota	**277**	225 (81.2)	28.9	19.1	52.0
Missouri	**405**	130 (32.1)	26.2	24.6	49.2
New Jersey	**491**	315 (64.2)	34.6	27.3	38.1
Tennessee	**573**	360 (62.8)	52.5	19.2	28.3
Utah	**548**	207 (37.8)	27.1	31.9	41.1
Wisconsin	**557**	219 (39.3)	44.7	20.5	34.7
**Total**	**5,058**	**3,007 (59.5)**	**35.2**	**23.1**	**41.7**
**Sex**					
Female	**945**	537 (56.8)^†^	35.6^¶^	25.3	39.1
Male	**4,111**	2,470 (60.1)	35.1	22.6	42.2
**Race/Ethnicity**					
White, non-Hispanic	**2,407**	1,402 (58.2)^§^	29.7**	22.7	47.6
Black, non-Hispanic	**1,041**	570 (54.8)	49.8	21.9	28.2
Hispanic	**1,019**	677 (66.4)	33.1	25.7	41.2

**Abbreviations:** ASD = autism spectrum disorder; IQ = intelligent quotient.

* Includes 25 children stated to have an IQ score in the average range but specific score was not given.

^†^ Pearson chi-square test for proportion of males versus females with ASD and IQ information (p = 0.07).

^§^ Pearson chi-square tests for proportion of Black, non-Hispanic versus White, non-Hispanic children with ASD and IQ information (p = 0.06); proportion of Black, non-Hispanic versus Hispanic children with IQ information (p<0.001); proportion of White, non-Hispanic versus Hispanic children with IQ information (p<0.001).

^¶^ Pearson chi-square test for proportion of males versus females with IQ ≤70 among children with ASD (p = 0.89).

** Pearson chi-square tests for proportion of Black, non-Hispanic versus White, non-Hispanic children with IQ ≤70 among children with ASD (p<0.001); proportion of Black, non-Hispanic versus Hispanic children with IQ information (p<0.001); proportion of White, non-Hispanic versus Hispanic children with IQ information (p = 0.13).

Among children aged 8 years with ASD who had data on cognitive ability, 35.2% were classified as having intellectual disability (IQ ≤70) at their most recent test or examination, 23.1% were classified in the borderline range (IQ = 71–85), and 41.7% were classified in the average or higher range (IQ >85) (Table 5). The percentage of children classified as having intellectual disability varied by site (range: 20.5% in California to 52.5% in Tennessee). Overall, the proportions of girls and boys classified as having an intellectual disability were similar (35.6% and 35.1%, respectively), and Black children were more likely than Hispanic and White children to be classified as having intellectual disability (49.8%, 33.1%, and 29.7%, respectively).

Previous ADDM Network reports included cognitive ability information only for sites collecting cognitive ability information on at least 60% of ASD cases. Applying that 60% threshold yielded similar percentages of children classified with intellectual disability overall, by sex, and by race and ethnicity (Supplementary Table 3, https://stacks.cdc.gov/view/cdc/111176).

### Age at First Evaluation and ASD Diagnosis

Among 4,681 children aged 8 years with ASD and recorded evaluations, 47.0% were evaluated by age 36 months (range: 40.6% in Tennessee to 66.4% in Maryland) (Table 6). The median age at first recorded evaluation ranged from 30 months in Maryland to 43 months in Missouri and Tennessee. Children with ASD and an IQ score ≤70 were more likely to be evaluated by age 36 months compared with children with ASD and an IQ score >70 (61.0% versus 45.5%). Age at earliest evaluation also was calculated using the same approach as previous ADDM Network reports, with similar findings (Supplementary Table 4, https://stacks.cdc.gov/view/cdc/111176).

**TABLE 6:** Number and percentage of children aged 8 years with autism spectrum disorder who received a comprehensive evaluation by a qualified professional at age ≤36 months, by site and intellectual disability status — Autism and Developmental Disabilities Monitoring Network, 11 sites, United States, 2018

Site	Total no. with ASD	Total with recorded evaluation			IQ ≤70			IQ >70		
		No. with recorded evaluation	% evaluated by age 36 mos	Median age at earliest recorded evaluation (mos)	No. with recorded evaluation	% evaluated by age 36 mos	Median age at earliest recorded evaluation (mos)	No. with recorded evaluation	% evaluated by age 36 mos	Median age at earliest recorded evaluation (mos)
Arizona	**331**	329	43.2	41	91	58.2	35	199	36.7	45
Arkansas	**353**	351	43.9	40	125	52.0	36	188	42.0	40.5
California	**586**	572	54.4	35	93	62.4	32	361	58.2	33
Georgia	**514**	445	48.8	37	136	60.3	34	217	43.8	42
Maryland	**423**	271	66.4	30	63	87.3	25	78	65.4	27
Minnesota	**277**	269	45.4	39	65	60.0	33	160	41.9	43
Missouri	**405**	400	40.8	43	34	41.2	43.5	94	25.5	60
New Jersey	**491**	490	46.1	39	109	45.9	37	206	51.5	36
Tennessee	**573**	534	40.6	43	181	66.9	32	163	30.7	49
Utah	**548**	494	43.3	42	55	49.1	39	148	42.6	43
Wisconsin	**557**	526	48.1	39	98	77.6	24	120	51.7	36
**Total**	**5,058**	**4,681**	**47.0**	**38**	**1,050**	**61.0**	34*	**1,934**	**45.5**	**39**

**Abbreviations:** ASD = autism spectrum disorder; IQ = intelligent quotient.

* Permutation test comparing median age of earliest known evaluation for children with known IQ score ≤70 versus known IQ score >70 (p<0.001).

Among the 3,833 children aged 8 years with ASD who had an evaluation containing an ASD diagnostic statement, the median age at earliest known diagnosis was 50 months (range: 36 months in California to 63 months in Minnesota) (Table 7). Children with ASD and an IQ score ≤70 had a lower median age at diagnosis (44 months) than children with an IQ score >70 (53 months).

**TABLE 7:** Median age at earliest known autism spectrum disorder diagnosis among children aged 8 years, by intellectual disability status — Autism and Developmental Disabilities Monitoring Network, 11 sites, United States, 2018

Site	Total no. with ASD	All children with an ASD diagnostic statement			Children with an ASD diagnostic statement and IQ score ≤70		Children with an ASD diagnostic statement and IQ score >70	
		No. with documented ASD diagnosis	Prevalence of ASD with documented diagnosis	Median age at earliest known diagnosis (mos)	No. with documented ASD diagnosis	Median age at earliest known diagnosis (mos)	No. with documented ASD diagnosis	Median age at earliest known diagnosis (mos)
Arizona	**331**	227	17.1	58	73	55	123	60
Arkansas	**353**	302	19.6	54	108	49	162	56
California	**586**	451	29.9	36	76	35.5	298	36
Georgia	**514**	321	13.6	52	110	46.5	139	60
Maryland	**423**	232	11.2	45	55	36	74	38.5
Minnesota	**277**	176	17.5	63	53	57	100	72
Missouri	**405**	371	15.2	51	31	54	89	74
New Jersey	**491**	462	26.7	45	103	45	196	44
Tennessee	**573**	426	16.9	53	159	34	132	61.5
Utah	**548**	394	15.5	54	41	47	113	59
Wisconsin	**557**	471	15.9	56	96	42	114	52
**Total**	**5,058**	**3,833**	**17.4**	**50**	**905**	44*	**1,540**	**53**

**Abbreviations:** ASD = autism spectrum disorder; IQ = intelligent quotient.

* Permutation test comparing median age of earliest known diagnosis for children with known IQ score ≤70 versus known IQ score >70 (p<0.001).

## Discussion

In 2018, ASD prevalence per 1,000 children aged 8 years varied across the 11 ADDM Network sites, ranging from 16.5 in Missouri to 38.9 in California. The overall ASD prevalence estimate was one in 44 children aged 8 years. These estimates are higher than ADDM Network ASD prevalence estimates from previous surveillance years. However, changing surveillance catchment areas over time can complicate analysis of trends. In 2018, the lowest ASD prevalence estimate was 16.5 per 1,000 children aged 8 years in Missouri, which was similar to the overall ASD prevalence estimate in 2014 (*9*). The ADDM Network is the only surveillance program in the United States that provides information about ASD in specific communities, including estimates for demographic subgroups. The variability across ADDM Network sites offers an opportunity to compare local policies and models for diagnostic and intervention service delivery that could enhance ASD identification and provide more comprehensive support to persons with ASD.

The California ADDM Network site had the highest ASD prevalence and the youngest median age at diagnosis and, as reported for early ASD identification for surveillance year 2018 (*16*), the cumulative incidence of early ASD identification at this site outpaced all other sites. California’s catchment area comprises a densely populated portion of metropolitan San Diego. Previous studies have found urban areas (*17*) and proximity to services (*18*,*19*) to be positively correlated with ASD prevalence. In addition, hundreds of pediatricians in San Diego have been engaged in a large research program to improve early ASD detection (*20*). California also has a system of regional centers that conduct assessments to determine eligibility for services for children with ASD. Previous studies estimated that these centers serve 75% of children with ASD in California and have demonstrated a temporal trend of decreasing mean ages at diagnosis over time (*21*,*22*). The local regional center was a contributing data source to the California catchment area. The contribution of these and other factors to early ASD detection in this community deserves further attention.

As in the 2016 surveillance year, no difference was observed in ASD prevalence among A/PI, Black, and White children aged 8 years overall. At multiple sites, ASD prevalence was lower for Hispanic children than for White or Black children, although the overall ASD prevalence for Hispanic children was similar to the other groups. Among the subsample of children with ASD and data on cognitive ability, a higher proportion of Black children than White or Hispanic children were classified with intellectual disability. This disparity has been reported previously (*7*–*10*). The reasons for this difference are not fully understood; however, they could overlap with factors such as preterm birth (*23*) and poverty (*24*) that contribute to, or are associated with, a higher overall intellectual disability prevalence among Black versus White children in the United States (*25*). Black children with ASD also have been reported to have less access to and use of services for ASD compared with White children with ASD (*26*). Although similar ASD prevalences across racial and ethnic groups might reflect equitable access to services, it is also possible that inequities in access to ASD diagnostic and treatment services persist. At two sites (Maryland and Minnesota), ASD prevalence was higher among Black children than among White children. If the actual prevalence of ASD is higher among racial or ethnic minority groups or among those who are economically disadvantaged, findings of similar prevalences across groups could mask ongoing disparities in access to ASD diagnosis and related services.

Data and research about AI/AN children with ASD are limited (*27*). The 2018 ADDM Network ASD prevalence estimate was 29.0 per 1,000 AI/AN children aged 8 years compared with approximately 21–22 per 1,000 in the other racial and ethnic groups; however, the sample size in the AI/AN group was limited. Monitoring ASD in one or more communities with sizable AI/AN populations would enable more meaningful comparisons with children in other racial and ethnic groups. AI/AN children with ASD might also have distinctive experiences accessing services, facing discrimination, and lacking culturally appropriate assessment tools (*27*). The Utah ADDM Network site has been involved in collaborative outreach activities with local AI/AN groups. Additional efforts are needed to better understand the unique situations and service needs of AI/AN children.

Socioeconomic status, measured by neighborhood-level MHI, was not consistently associated with ASD prevalence across sites. This contrasts with previous analyses of the ADDM Network data reported for surveillance years 2002–2010 indicating a robust positive association between ASD prevalence and SES (*28*,*29*). Studies of special education data from the United Kingdom (*30*) and administrative data from California (*31*) and Sweden (*32*) have reported that children in low-income households or living in lower SES neighborhoods are more likely to be identified as having ASD than are children from higher SES neighborhoods. In addition, the new ADDM Network case definition could be more likely than the previous one to include children of lower SES because the previous case definition excluded children without sufficiently detailed records (*33*). Multiple states have programs to serve children with ASD from low-income households and insurance mandates to cover ASD services. For example, “Learn the Signs. Act Early.” focuses on inclusion of developmental monitoring resources for families with low incomes, such as those served by Early Head Start, Head Start, and the Supplemental Nutrition Program for Women, Infants, and Children (https://www.cdc.gov/ncbddd/actearly/wic-providers.html). Further analyses, including all surveillance years since 2010 and additional measures of SES and confounding factors, are warranted.

Many children had multiple evaluations with inconsistent findings (e.g., ASD was suspected or ruled out before being confirmed). Certain children were identified as having ASD only in a clinic or at school but not both. Similar to variability in the timing and type of ASD identification among sites, the proportion of children in whom ASD was ruled out before being confirmed varied by site and did not appear related to ASD prevalence. The two sites with the highest ASD prevalence estimates (California and New Jersey) had the highest and lowest proportions, respectively, of children with ASD but with a history of ASD being ruled out. Further analyses of these data might help in understanding the barriers, delays, and conflicting information many families experience during the process of ASD diagnosis and as they attempt to connect with services for ASD (*34*). Specialized training and diagnostic tools often are often recommended for the assessment of ASD (a definitive diagnostic biomarker is not available) (*35*), and criteria for classifying ASD vary across states and systems. Certain states, including New Jersey, require an assessment by a physician trained in neurodevelopmental assessment to assign an ASD classification in special education (*36*), whereas others do not. Comparisons of states’ ASD-related policies or requirements for ASD services also could enhance interpretation of these findings.

Most of the data collection and record reviews for the ADDM Network surveillance year 2018 were conducted during the COVID-19 pandemic. Record reviews were limited at three sites because of physical access restrictions and a lack of remote or electronic access (Table 1). These restrictions resulted in less complete data for items that required manual chart review (e.g., evaluations, documented ASD diagnoses, and cognitive and adaptive tests). Children could meet the ASD case definition without a full record review if ASD ICD codes or ASD special education exceptionalities were initially transmitted by the data sources to the ADDM Network site. However, any indications of ASD that would only be available through a manual record review, such as ASD diagnostic statements in evaluations, would not be captured if a manual record review could not be completed, resulting in likely underascertainment of ASD cases. Overall, approximately 6% of ASD cases among children aged 8 years were only ascertained through a manual record review, although this percentage varied by site (Supplementary Table 2, https://stacks.cdc.gov/view/cdc/111176).

The ADDM Network used a new case definition and data collection process for surveillance year 2018. The previous ASD case definition was based on operationalized criteria described in *DSM-5* and involved detailed abstraction and expert clinical review of behavioral symptoms documented in children’s evaluations (*1*). An analysis using data from ADDM Network surveillance years 2014 and 2016 compared the case definitions and found that, compared with the overall ASD prevalence using the previous case definition, ASD prevalence using the new case definition was approximately the same for 2014 and 7% lower for 2016 (*12*). Other indicators, such as prevalence ratios, ages at evaluation or ASD diagnosis, and co-occurring intellectual disability, were similar using both case definitions. Approximately 86% of all children who met either the previous or new case definition met both case definitions. The new case definition did not ascertain ASD among children who were never identified as having ASD by a community provider. Conversely, the previous case definition excluded certain children who had been identified as having ASD by a community provider because the records lacked sufficiently detailed clinical information to confirm the diagnosis. Possibly, certain sites could have reported higher ASD prevalence using the previous case definition, although the new case definition enabled ASD to be ascertained from new data sources (such as Medicaid-funded ASD services) at certain sites and was more robust when children’s evaluations were not accessible (such as during the COVID-19 pandemic).

## Limitations

The findings in this report are subject to at least six limitations. First, the methods rely on the availability and completeness of existing information and records to ascertain ASD cases and other indicators. Two sites (Missouri and Wisconsin) lacked access to education data sources for large portions of their population and might not ascertain ASD cases among children who only receive services for ASD at school. Incomplete information could lead to misclassifying children’s cognitive ability, overestimating the age when they were first evaluated or when ASD was diagnosed, or failing to ascertain that the children were identified as having ASD. Similarly, the records of more than one third of children with ASD were missing IQ scores or other measures of cognitive ability. The completeness and availability of data could contribute to variability across sites, and children who were administered cognitive tests might differ from those who were not. Second, cognitive ability was measured on the basis of a child’s latest cognitive test or examiner statement of a child’s cognitive ability. IQ scores are not necessarily stable measures of intellectual ability over time, can increase substantially in children with ASD in response to intensive early therapeutic interventions (*37*), and might be especially unstable during early childhood (*38*). The age at which children had their most recent test or examiner impression of cognitive ability varied by site. Third, sites participating in the ADDM Network are selected through a competitive process, and the resulting catchment areas are not designed to be representative of the states in which the sites are located. Findings do not necessarily generalize to all children aged 8 years in the United States, and interpretations of temporal trends are complicated by changing catchment areas, case definitions, and diagnostic practices. Fourth, small numbers result in imprecise estimates for certain sites and subgroups. Fifth, the surveillance data system does not record the number of times a child received an ASD ICD code at a specific source. In a future analysis, it might be possible at certain sites to examine the number of times children received ASD ICD codes or the extent to which reporting errors in ICD codes occurred among the 5.4% of children for whom ICD codes are the only indicator of ASD. Finally, validation studies are needed to estimate undiagnosed ASD as well as false-positive diagnoses.

## Future Directions

For the 2020 surveillance year, the ADDM Network continued data collection to monitor ASD prevalence among children aged 4 and 8 years with the same 11 sites. Therefore, it might be possible to assess changes or disruptions in evaluations or services caused by the COVID-19 pandemic. For surveillance years 2018 and 2020, five of the 11 sites collected information on children aged 16 years whose ASD cases were initially ascertained by ADDM Network surveillance at age 8 years. Seven of the ADDM Network sites conducted a pilot program of a low-cost statewide surveillance approach intended to estimate ASD prevalence at the county level by linking electronically available data (i.e., no manual record review). Standard demographic categories were adopted for surveillance year 2020 that documented race and ethnicity separately and included a multiracial category in the population denominator. This change allowed the ADDM Network to distinguish AI/AN children who are also Hispanic (previously coded only as Hispanic). Future analyses (potentially through more extensive data linkages) might be able to portray disparities more directly related to the receipt of specific ASD-related interventions or support.

## Conclusion

Findings from the ADDM Network 2018 surveillance year highlight the variability in ASD prevalence and identification practices across communities and report an overall higher ASD prevalence than previous estimates from the ADDM Network. Research into the factors associated with the variability in ASD prevalence across communities and the higher proportion of intellectual disability among Black children with ASD is warranted. Progress is still needed in certain important areas, including the lower identification of ASD among Hispanic children versus other demographic groups. Evidence exists of persistent disparities for various subgroups. These findings emphasize the need for sustained efforts to reduce geographic, racial, and ethnic disparities in identification of and support for persons with ASD.
